# Constancy and Change: Key Issues in Housing and Health Research, 1987–2017

**DOI:** 10.3390/ijerph14070763

**Published:** 2017-07-12

**Authors:** Roderick J. Lawrence

**Affiliations:** 1 University of Geneva, 1211 Geneva 4, Switzerland; Roderick.lawrence@unige.ch; Tel.: +41-22-3790872; 2 Institute for Environment & Development (LESTARI), Universiti Kebangsaan Malaysia, 43600 Bangi, Selangor, Malaysia; 3 School of Architecture & the Built Environment, University of Adelaide, 5005 Adelaide, Australia

**Keywords:** applicability gap, coproduction, interdisciplinary, public policies, transdisciplinary

## Abstract

The low impact of scientific research on the relations between housing and health during the last 30 years can be attributed to a number of reasons. First, statistical analyses have meant to improve understanding of the relations between what are interpreted and measured as causal factors. However, any single statistical approach fails to account for the dynamic non-linear relations between multiple factors and therefore cannot analyze systemic complexity. Second, there has been too little accumulation and validation of knowledge from scientific research owing to the dominance of cross-sectional studies, and the lack of coordinated research agendas using these approaches in order to confirm empirical findings. Hence, there is little evidence indicating that public policies in both the housing and the public health sectors in specific localities have benefited from the accumulated evidence of empirical research. Third, the findings from empirical studies have been published in academic journals and monographs but rarely disseminated to actors and institutions in the public and private sectors. Hence housing and health research and policy formulation have not been consolidated during the last three decades. The author of this communication argues for a radical shift from conventional disciplinary and multi-disciplinary contributions to transdisciplinary research programmes and projects that formulate and apply innovative approaches founded on conceptual frameworks that apply systems thinking for the integration of knowledge and know-how of researchers, policy makers, and professional practitioners in precise localities.

## 1. Introduction

During the last 30 years, numerous research projects, publications and conferences on housing and health have been achieved in many countries around the world. Some institutions and associations including the European Network for Housing Research (ENHR) have formed working groups that discuss research on this subject [1]. Scientific and professional journals have published special issues on housing and health [2,3]. The World Health Organization Regional Office for Europe founded a taskforce on housing and health in 2002. One outcome has been a Large-Scale Pan-European Survey (LARES) of housing and health completed in eight European cities in 2003 [4]. Housing and health was a key theme addressed by delegates at the European Ministerial Conference on Environment and Health in Budapest in June 2004. Then, in March 2010, delegates at the Fifth European Ministerial Conference on Environment and Health, held in Parma from 10 to 12 March 2010, considered actions to address environmental health risks in housing and residential neighbourhoods that impact on children. An enlarged interpretation of these risks included the influence of urban design projects and land use planning on the increasing incidence of obesity in children. Then, the World Health Organization hosted an international consultation in Geneva from 13 to 15 October 2010. The 40 delegates from 18 countries agreed that housing and residential environments are localities having high environmental health risks in relation to injury from accidents, as well as infectious and non-communicable diseases. The delegates recommended that guidelines on “healthy housing” should be developed to promote preventive measures through better housing [5].

All of these contributions share a common concern to improve our understanding of the multiple relations between diverse characteristics of housing environments that can positively or negatively influence physical and mental health, and the social wellbeing of individuals, households, and population groups. Despite these good intentions, the impact of empirical research and policy dialogue has not been effective in accumulating, validating and applying knowledge that could improve housing and health in all regions of the world. Today, there still is a disconnection between the findings of scientific research on housing and health, normative public policies in the health and housing sectors, and the contextual knowledge required to promote health in precise localities [6]. This disconnection should be overcome in order to formulate more effective interventions across multiple levels of the built environment (from housing units to streets, neighbourhoods and cities).

The following sections of this communication discuss the reasons for these ineffective outcomes. First, statistical research has meant to improve understanding of the relations between what are interpreted and measured as causal factors. However, any single statistical approach fails to account for the dynamic, non-linear relations between multiple factors; therefore, this method cannot analyze the systemic complexity of housing and health. Research of this kind has not identified and studied those context-dependent factors, or the multiple relations between them, prior to interventions that are meant to improve the health of residents in precise situations. Second, there has been too little accumulation and validation of knowledge from scientific research owing to the dominance of cross-sectional studies, and the lack of coordinated research agendas; therefore, these kinds of contributions rarely confirm the empirical findings of other studies in specific localities. Indeed, there are distinctly different, sometimes competing, interpretations of housing and health that have been published. Hence policy makers and practitioners have great difficulty in understanding this complex subject. The accumulation and validation of information and knowledge has not been as important as it could have been [7]. Consequently, there is little evidence indicating that public policies in both the housing and the public health sectors in specific localities have benefited from the cumulative findings of empirical research. Third, while there have been many scientific publications in peer reviewed journals during the last 30 years, dissemination of research results to nonacademic actors and institutions in the public and private sectors has not been a high priority. Hence the findings from empirical studies have been published in academic journals and monographs but rarely disseminated to professionals in the public and private sectors. Consequently, collaboration between housing and health researchers and policy decision makers has not been consolidated during the last three decades. This outcome corresponds to the period when public investments in the housing sector decreased in all Organization for Economic Co-operation and Development (OECD) member countries [8].

In order to increase the impact of contributions in this field, there is a need for a radical shift from disciplinary and multidisciplinary research (that considers only selected discipline based factors) to transdisciplinary programmes and projects that formulate and apply innovative approaches by creatively using the transfer of knowledge and know-how between researchers, policy makers and professional practitioners. The next section of this communication underlines the challenge of dealing with real-world problems concerning housing and health. There is a growing volume of empirical information and knowledge that this challenge extends well beyond the conventional housing and health sectors. This communication argues that both housing and health are not just disciplinary subjects of professional expertise; they are fundamental societal challenges that require collective understanding and action.

## 2. Beyond Disciplinary Boundaries

Housing should accommodate and facilitate basic human needs for shelter and security of the occupants against climatic conditions (excessive heat and cold) and harmful intrusions from insects, rodents and environmental nuisances, such as noise, that may impact negatively on health and wellbeing [9]. Housing also accommodates household activities and possessions. Housing should also be interpreted as a set of processes and outcomes including the provision and maintenance of all kinds of residential buildings, either by public authorities or private initiatives. The right to habitable and affordable housing is included in article 25.1 of the Universal Declaration of Human Rights adopted in 1948 [10].

The housing environment can also be considered in terms of its capacity to nurture and sustain individual and collective human processes [11]. For example, the capacity of any resident in her/his domestic environment to alleviate fatigue or stress, and whether this restorative process is mediated by views of nature, or time spent in natural surroundings such as urban parks [12]. In addition, the material standard of housing units should be considered in relation to forms of housing tenure, household composition and income, the availability and cost of construction materials, infrastructure and services, the education level of residents, and the employment status of heads of households. These multiple dimensions of housing environments and the health of residents should not be isolated from their diet, lifestyle, employment status and the availability of health care. Hartig and Lawrence coined the term “the residential context of health” to refer to all these variables and their interrelated nature [13].

During the last 30 years, numerous empirical studies have identified and measured the relations between the specific characteristics of housing and health outcomes. Fortunately, there has been a widening of scope of scientific research: For example, a common assumption in the 1970s that floor level above the ground of housing units correlated with adverse effects on mental health has been corrected and qualified by the application of concepts in the field of people-environment studies, such as choice in housing markets, individual preferences, tenure, and residential mobility [14]. In a similar vein, the study of damp, mould and affordable warmth analysed in the 1980s in relation to excess mortality during winter months has been complemented by recent concerns about energy consumption, carbon dioxide emissions and climate change [15]. This broader scope provides the context for other studies of ambient temperatures in summer months and excess mortality during and after heat waves [16].

Quantitative research methods have dominated the field of housing and health during the last 30 years. Many authors have applied statistical analyses using regression-based methods. These statistical analyses are meant to improve understanding of the quantifiable relationship between what authors identify and interpret as causal factors. As Kreiger noted, causal inference in empirical research on housing and health is a challenge owing to the systemic complexity of the subject [17]. Any statistical approach fails to account for the multiple characteristics of dynamic, non-linear and reciprocal relations between pertinent factors that are context dependent [18]. Consequently, Baker and colleagues propose and implement the study of a “bundle of factors” in their contribution to this special issue [19].

These and many other contributions confirm that when housing and the built environment are considered only from a specific disciplinary perspective then the multiple relations between housing, health and wellbeing may not be considered as important. For example, if housing units are constructed such that their material conditions only meet minimum building standards, then the social and psychological dimensions of the home environment will probably be overlooked to the detriment of the mental health and wellbeing of the residents [20]. There is a growing recognition of a need for innovative approaches in the field of housing and health.

The next section of this communication outlines a way forward that has been increasingly applied in the domains of the built environment and public health since the 1990s. Innovative contributions require a fundamental rethinking of the nature of housing and health; systems thinking about the interrelated nature of all the constituents that should be addressed; the need for analysis of these constituents at different levels from individual rooms in the housing unit to the residential neighbourhood and beyond. These methodological issues are not only the responsibility of researchers but also policy makers and professionals in both the housing and health sectors. However, it has been extremely rare for explicit collaboration between researchers, policy makers and practitioners in both these domains [21]. This is an important challenge that should be addressed in the near future.

## 3. Looking Ahead

Today, we know that the multiple and complex relations between housing and health are not confined to traditional disciplinary and professional boundaries. This knowledge should be applied to implement a significant change from conventional disciplinary and multidisciplinary research to innovative interdisciplinary and transdisciplinary contributions using a range of quantitative and qualitative research methods. This shift can provide the foundation for participative research and professional practice as some innovative contributions have shown [22].

### 3.1. Complex Systems: Housing and Health

Systems theory rejects determinism and reductionism that have led to studies of individual components of housing systems and health systems isolated from their societal context (see above). Systems theory posits that causation is non-linear and that minor changes in one part of a system can have larger effects elsewhere in that system. Housing and health should be interpreted as a complex system related to others (e.g., mobility and transport infrastructure) in real-world situations. The housing-health system comprises many proximate and distal components that interact mutually at these different levels. These systems should not be studied using linear interpretations of cause–effect models because such simplification cannot represent extant situations. Research of variables isolated from their context should be replaced by case based studies [23]. Although contemporary public health challenges, including urban health, are increasingly recognized by researchers and funding agencies as systems with emergent properties, in general, systemic approaches have been used to describe and model real world situations rather than define, monitor and evaluate interventions. Research institutes and funding agencies should promote a reorientation of action research by allocating specific funds for this kind of case based interventions.

### 3.2. Moving from Interdisciplinary to Transdisciplinary Contributions

Interdisciplinary contributions have been implemented in the natural and social sciences for about a century. These contributions involve explicit collaboration between several disciplines in order to advance scientific knowledge about specific subjects. Each discipline retains its own concepts and methods that are applied to a mutually agreed subject. Here, it is worth recalling the origins of the 19th century public health movement and strong interventions by philanthropic institutions and municipal services in the housing sector. These interventions involved collaboration between architects, engineers, housing reformers, medical practitioners, politicians and public health officers to deal with a societal challenge at that time. More recently, the example of Intimate Partner Violence (IPV) has been used by O’Campo and colleagues to show the added value of a broad, interdisciplinary understanding of this public health challenge [24]. Collaboration between researchers from different disciplines may provide a synergy for the development of new concepts. One example is the concept of resilience initially formulated in physics, then transferred to the medical and health sciences, the social sciences and psychology [25]. Lawrence argued that interdisciplinary contributions highlight the difference between a discipline-based interpretation of housing and health and others that reject an explanation based only on a bio-medical model of health in order to analyse the compound influence of multiple human and extra-human factors [26].

In contrast to interdisciplinary research, the defining characteristic of transdisciplinary contributions is transcendence. These kinds of contribution cross the boundaries of scientific knowledge in order to account for other types of knowledge (scientific knowledge, professional know-how and tacit knowledge). Hence transdisciplinary contributions create a broader knowledge domain than interdisciplinary contributions; they are founded on the coproduction of knowledge by actors and institutions for socially accepted projects that are meant to impact on real world situations [27]. The context-dependent nature of knowledge and societal action are fundamental. In the case of housing and health, information and knowledge from scientific research is complemented by the non-academic knowledge and know-how of policy makers and professionals in both sectors, as well as the tacit knowledge of community based individuals and institutions. Transdisciplinary contributions involve mutual learning, joint problem definition, agreement on the allocation of resources, and the sharing of data and information about precise situations [28]. Consequently, the limitations of normative contributions that were common in the 20th century need to be recognized and corrected.

There are a growing number of examples of transdisciplinary projects, especially pragmatic responses to housing and urban situations that involve a number of non-academic actors and institutions. These contributions are meant to reduce the long-standing applicability gap between research and professional practice [29]. This means that communication and negotiation processes between participants in transdisciplinary projects are crucial to ensure the credibility, legitimacy and salience of selected subjects and research questions. In this respect, transdisciplinary contributions are quite different from basic research, interdisciplinary research and applied research [30].

## 4. Discussion

Collaborative research involving researchers trained in different disciplines was marginal in the field of housing and health until the 1980s. However, the tendency for researchers to work within disciplinary boundaries has evolved but not significantly since then. Despite arguments for transdisciplinary research in the field of public health, and housing and health in particular, these kinds of contributions have still not become mainstream. There are academic and institutional reasons for this that should be addressed by public and private institutions in higher education as well as funding agencies [31]. These obstacles extend beyond the choice of disciplinary confinement by many individuals concerned, above all, about recognition by colleagues and career promotion in their own discipline (especially in the medical and health sciences). These obstacles also concern the departmental organisation and structure of universities founded on strong disciplinary borders (to the detriment of cross-fertilization between disciplines), and the attention to academic publication channels that prioritize specific competences (at the expense of team science and collaborative research), as well as the relatively low allocation of public and private funds for interdisciplinary research (which are still not commonly prioritized by research funding agencies). Fundamental changes in institutional arrangements, including the allocation of specific funds for inter- and trans- disciplinary research, have occurred in the last two decades. Also, some innovative training programs in interdisciplinary research, systems thinking and collaborative decision making have been proposed. However, in general, administrative, institutional and financial obstacles still outweigh incentives for innovative research.

Given that the interrelations between people and their immediate surroundings should not be delimited and reduced to traditional disciplinary concepts and methods, more interdisciplinary and transdisciplinary contributions are necessary in order to address the complexity of housing and health and implement effective responses to real-world situations. These kinds of contributions can apply a broad integrated perspective which should be taught in institutions of higher education and professional training. This perspective is still not widely understood by professions in the public health sector, and others in the housing and planning sectors.

This author has noted elsewhere that current debate on disciplinary versus interdisciplinary research discussed in the Special Issue of *Nature* (16 September 2015) needs to be reconsidered. He has explained why “disciplinary, interdisciplinary and transdisciplinary research can and should coexist, because the co-benefits of transdisciplinary research for individuals, research groups, and research institutions in the public and private sectors can lead to added value for society” [32]. This is a challenging way forward for the field of housing and health and indeed other public health challenges that should be confronted [33].

## 5. Conclusions

The shift in the definition and implementation of research about housing and health requested in this communication reflects a growing concern about the limitations of conventional knowledge production (that is autonomous, discipline based, hierarchical, and grounded on academic criteria) in order to address the knowledge–action interchange in both the housing and health sectors. Studies of housing and health should not only be motivated by the curiosity of scientific researchers but also driven by societal concerns that account for the viewpoints of actors and institutions effected by the specific subject and situation of mutual concern.

Transdisciplinary knowledge production moves beyond conventional research agendas to address real world concerns not confined to disciplinary competences and professional know-how. These kinds of contributions are not only driven by the curiosity of academics or other researchers. They are meant to address fundamental societal challenges in many domains that require collective understanding, political commitment, and innovative responses. Unfortunately, today there is no shared understanding about an interdisciplinary and a transdisciplinary epistemology of housing and health. The formulation and application of shared conceptual and methodological frameworks should be an objective of this field of inquiry in the immediate future.
